# Single-Nucleotide Polymorphisms and Markers of Oxidative Stress in Healthy Women

**DOI:** 10.1371/journal.pone.0156450

**Published:** 2016-06-07

**Authors:** Albina N. Minlikeeva, Richard W. Browne, Heather M. Ochs-Balcom, Catalin Marian, Peter G. Shields, Maurizio Trevisan, Shiva Krishnan, Ramakrishna Modali, Michael Seddon, Teresa Lehman, Jo L. Freudenheim

**Affiliations:** 1 Department of Epidemiology and Environmental Health, University at Buffalo, Buffalo, New York, United States of America; 2 Department of Biotechnical and Clinical Laboratory Sciences, University at Buffalo, Buffalo, New York, United States of America; 3 The Ohio State University Comprehensive Cancer Center, Division of Cancer Prevention and Control, Columbus, Ohio, United States of America; 4 Victor Babes University of Medicine and Pharmacy, Timisoara, Romania; 5 Sophie Davis School of Biomedical Education, City College of New York, New York, New York, United States of America; 6 BioServe Biotechnologies Ltd., Beltsville, Maryland, United States of America; Instituto de Higiene e Medicina Tropical, PORTUGAL

## Abstract

**Purpose:**

There is accumulating evidence that oxidative stress is an important contributor to carcinogenesis. We hypothesized that genetic variation in genes involved in maintaining antioxidant/oxidant balance would be associated with overall oxidative stress.

**Methods:**

We examined associations between single nucleotide polymorphisms (SNPs) in *MnSOD*, *GSTP1*, *GSTM1*, *GPX1*, *GPX3*, and *CAT* genes and thiobarbituric acid-reactive substances (TBARS), a blood biomarker of oxidative damage, in healthy white women randomly selected from Western New York (n = 1402). We used general linear models to calculate age-adjusted geometric means of TBARS across the variants. We also examined the associations within strata of menopausal status.

**Results:**

For *MnSOD*, being heterozygous was associated with lower geometric means of TBARS (less oxidative stress), 1.28 mg/dL, compared to homozygous T-allele or homozygous C-allele,1.35 mg/dL, and 1.31 mg/dL correspondingly (p for trend = 0.01). This difference remained among postmenopausal women, 1.40 mg/dL for TT, 1.32 mg/dL for TC, and 1.34mg/dL for CC (p for trend 0.015); it was attenuated among premenopausal women. SNPs in the other genes examined (*GSTP1*, *GSTM1*, *GPX1*, *GPX3*, and *CAT*) were not associated with TBARS.

**Conclusions:**

Our findings suggest that genetic variation in *MnSOD* gene may be associated with oxidative status, particularly among postmenopausal women.

## Introduction

Reactive oxygen species (ROS) are by-products of metabolism which, in excessive amounts, can initiate and promote carcinogenesis [1–3]. To manage the damaging effects of ROS, their concentrations are controlled by the endogenous antioxidant defense system [4] that include antioxidant enzymes (AOE) such as manganese superoxide dismutase (MnSOD), glutathione peroxidases (GPx), catalase (CAT), and glutathione-S-transferases (GSTM1, GSTP1) [5, 6]. MnSOD, GPX1, GPX3, and CAT are involved in deactivation of the superoxide anion radical and downstream ROS including hydrogen peroxide [3, 5]. GSTM1 and GSTP1 enzymes constitute a second line of defense [5], which can detoxify electrophilic compounds that can cause oxidative stress [7].

The activity of the AOEs is affected by single nuclear polymorphisms (SNPs) in these genes [7–11]. Change in the activity of AOEs’ impacted by corresponding SNPs may influence oxidative stress [12]. There have been numerous studies focused on the association between the SNPs and risk of various pathological conditions associated with increased oxidative stress, particularly cancer [13–23]. However, very few studies [12, 24] have been conducted to examine the relationship between genetic variation in these enzymes and oxidative stress biomarkers, specifically among healthy individuals.

Thiobarbituric acid-reactive substances (TBARS) is a biomarker of oxidative stress. The TBARS spectrophotometric assay detects the concentration of a chromogen produced as a result of a reaction between thiobarbituric acid and a product of lipid peroxidation, malondialdehyde (MDA)[25]. MDA has been widely used as a marker of oxidative stress [26]; for example, it has been shown to be elevated among patients diagnosed with cancer compared to healthy controls in some studies [27–29].

We examined the association between SNPs in *MnSOD*, *GSTP1*, *GSTM1*, *GPX1*, *GPX3*, and *CAT* genes and TBARS in a randomly selected population-based sample of healthy women. We hypothesized that SNPs in genes involved in maintaining antioxidant/oxidant balance influence oxidative stress altering TBARS concentration.

## Materials and Methods

The Western New York Exposures and Breast Cancer (WEB) study is a population-based case-control study of breast cancer conducted between 1996 and 2001 [30]. The WEB study included 2,115 healthy women as controls; data from the control women were used for this analysis. Briefly, the women were 35 to 79 years of age residing in Erie and Niagara counties of the state of New York, were fluent in English and had no history of cancer other than non-melanoma skin cancer. Those less than 65 years of age were identified from the Department of Motor Vehicles driver’s license lists; those ages 65 and older were randomly selected from the rolls of the Health Care Financing Administration. Among eligible controls, 63% agreed to participate in the study and provided informed consent. The protocol was reviewed and approved by the Institutional Review Board of the University at Buffalo.

Participants completed comprehensive self-administered questionnaires and in-person interviews conducted by trained personnel. The data collected included demographics, smoking history and smoking status on the date of interview, and alcohol consumption, use of hormone replacement therapy, and non-steroidal anti-inflammatory drugs 12 to 24 months prior to interview. Body mass index was calculated based on height and weight measured during the interview by trained interviewers using a standardized protocol and expressed in kg/m^2^.

Dietary intake of vitamins C and E were derived from a self-administered modified version of the Health Habits and History food frequency questionnaire with the DietSys nutrient analysis software (version 3.7). Dietary and supplement intake of the vitamins during 12 to 24 months prior to interview were used to obtain a total daily intake for each vitamin.

### TBARS measurement

Blood for the analysis was drawn by trained phlebotomists on the morning of the interview, between 7:30 am and 9:30 am, after at least 12 hours of fasting and at least 30 minutes after smoking and physical exercise. For premenopausal women, the blood draw was done during the luteal phase of menstrual cycle.

Plasma for TBARS assay was collected into 15%K3EDTA anticoagulant to chelate divalent metal ions and prevent fenton type reactions. Samples were immediately wrapped in aluminum foil to protect them from light, placed on wet ice, transferred to the specimen processing laboratory and processed within 2 hours of collection [31].To prevent autooxidation during TBARS assay, plasma samples (1 mL) were spiked with 10 uL of a 5% solution of Butylated hydroxytoluene in methanol prior to analysis. The processed aliquots were then frozen at -80°C. Plasma (15% K3 EDTA) TBARS, expressed in nmol/ml of malondialdehyde equivalents, were measured in duplicate by the method of Armstrong and Browne [32]. Calibration standards and quality control (QC) samples were included in every run. QC samples were run in duplicate and included three levels of “in-house” pooled human EDTA plasma, stored in aliquots at -80°C [33]. QC ranges were established from 20 determinations in the same run and five samples per day over a period of 20 days. Levey-Jennings QC plots were employed to track QC data across all participant samples and Westgard multirules were used to assess run acceptability. The intra-and interassay coefficients of variation for TBARS were 7.6% and 9.2% respectively across the study [34].

### Genotyping

Blood was drawn from all consenting participants who agreed to provide samples (88% of controls). The DNAQuik^™^ (BioServe, Beltsville, MD) extraction kit was used to obtain DNA from blood cells.

We examined the following SNPs: rs4880 for *MnSOD*, rs1695 for *GSTP1*, rs1050450 for *GPX1*, rs1946234 for *GPX3*, and rs1001179 for *CAT* genes. The SNPs for these analyses were selected based on functional impact on the coded protein including variants which change the sequence or expression of the protein. To determine the GSTP1_rs1695 genotype, the real-time polymerase chain reaction (RT-PCR) Taqman allelic discrimination with pre-designed assays (Applied Biosystems, Foster City, CA, USA) was utilized. The procedure was done according to the manufacturer’s instructions. Genotyping of MnSOD_rs4880 (primers 5-ACGTTGGATGCTGTGCTTTCTCGTCTTCAG-3’ and 5’-ACGTTGGATGTTCTGCCTGGAGCCCAGATA-3’), GPX1_rs1050450 (primers 5’-ACGTTGGATGATCCCGAGACAGCAGCA-3’ and 5’- ACGTTGGATGATCGAGCCTGACATCGAAGC), GPX3_A13870C_rs1946234 (primers 5’- ACGTTGGATGTGTTGCAGTTACTCTCTGGG-3’ and 5’-ACGTTGGATGACAAGTCATCTAGCCAGAGC-3’), and CAT_rs1001179 (primers 5’- ACGTTGGATGAGCAATTGGAGAGCCTCGC-3’ and 5’-ACGTTGGATGCTGAAGGATGCTGATAACCG-3’) was done utilizing polymerase chain reaction (PCR) followed by genotyping using the MassARRAY iPLEX^™^ platform (Sequenom).The sequences of the primers were designed by utilizing SpectroDESIGNER software (Sequenom), and the primers themselves were synthesized at BioServe Biotechnologies, Ltd (Beltsville, MD). The Homogenous Mass Extend reaction was used to prepare the PCR products for matrix-assisted laser desorption ionization-time of flight mass spectrometry (MALDI-TOF MS) [35]. Genotyping of *GSTM1* was done utilizing TaqMan realtime PCR with two 5-nuclease probes and four PCR primers according to the method described by Moore et al. [36]. For GSTM1, the presence of deletion was determined.

Quality control procedures were identical for all SNPs and were performed for each laboratory assay and included positive and negative controls in all runs and samples analyzed as blind duplicates (20%). All concordance rates were at least 91.7%, and call rates were above 93.7%.

### Statistical analysis

Of 2,115 women in the study, there were 575 (508 white and 67 non-white women) with missing information on at least one genotype or a TBARS result, and so were excluded from these analyses. Genotype distributions for each SNP (except for *GSTM1*) were tested for Hardy-Weinberg equilibrium for the total group and the participants stratified by race (whites and non-whites). Hardy-Weinberg equilibrium was violated for the *CAT* genotype among non-white individuals (n = 138); to make the analysis more consistent, analyses were limited to whites. Those who remained were less likely to be users of ibuprofen (42.4% vs.50.6%) and more likely to be premenopausal (31.9% vs.25.8%) compared to the white participants excluded on the basis of missing information on the exposures or outcome.

We examined the correlation between TBARS and age with the Spearman correlation. Using general linear models, we calculated age-adjusted geometric means of TBARS across all the variants. Additional adjustment for other factors which might be related to oxidation including menopausal status, HRT use, number of hours of physical activity over the past seven days, smoking status, secondhand smoke exposure, body mass index, alcohol consumption, aspirin/ibuprofen consumption, education, and total intakes of vitamins C and E did not change the estimates for more than 10%. These variables were not included in the final models.

We obtained the p-values for trend tests by modeling genotypes as continuous variables.

We also examined effect modification by menopausal status, exploring associations separately for pre- and postmenopausal women.

The analyses were conducted using SAS 9.3 software. All the statistical tests were two-sided, and level of significance was 5%.

## Results

Demographic characteristics of the participants are presented in Table 1. Women were more likely to be postmenopausal, never users of hormone replacement therapy, never/former smokers, current drinkers, and non-users of aspirin. Concentration of TBARS was significantly associated with age, but the association was not strong (Spearman correlation 0.20, p<0.001).

**Table 1:** Characteristics of the participants, WEB study controls, Erie and Niagara Counties, 1996–2001^a^.

Characteristics	All participants, n = 1402
Age (years)	56.6 (11.5)
Body mass index (kg/m^2^)	27.9 (6.3)
Menopausal status	
Premenopausal	447 (31.9)
Postmenopausal	955 (68.1)
Use of hormone replacement therapy	
Never	868 (64.1)
Ever	486 (35.9)
Education (years)	13.4 (2.3)
Physical activity over the past 7 days (hours)	8.4 (10.1)
Smoking status	
Never/former smoker	1193 (85.3)
Current smoker	206 (14.7)
Alcohol consumption	
Lifetime/not current drinker	669 (48.2)
Current drinker	720 (51.8)
Aspirin use	
Non-user (0 days/month)	767 (55.1)
Infrequent user (≤14 days/month)	454 (32.6)
Regular user (>14 days/month)	170 (12.2)
Ibuprofen	
Non-user (0 days/month)	590 (42.5)
Infrequent user (≤14 days/month)	689 (49.6)
Regular user (>14 days/month)	111 (7.9)
Total daily vitamins intake	
Vitamin C (mg)	174.1 (106.3–388.5)
Vitamin E (IU)	34.3 (8.9–221.4)
TBARS^b^ (nmol/ml)	1.3(1.1–1.5)

^a^Data shown are means (SD) for continuous variables; n (%) for categorical variables, and values of median (interquartile range) for the total daily intake of vitamins C and E and for TBARS concentration.

^b^TBARS- Thiobarbituric acid-reactive substances

Blood concentration of TBARS was associated with *MnSOD* genotypes (Table 2). In the overall sample, the age-adjusted geometric mean of TBARS was highest among those with the TT genotype, 1.35 nmol/ml (p for trend = 0.01). After stratification based on menopausal status, for postmenopausal women the value of the age-adjusted geometric mean of TBARS remained the lowest among heterozygous individuals, 1.32 nmol/ml, compared to either TT- or CC-genotypes, 1.40 nmol/ml and 1.34 nmol/ml correspondingly (p for trend = 0.015). For premenopausal women, the geometric means of TBARS concentration did not differ considerably by *MnSOD* genotype, 1.25 nmol/ml for those with TT genotype, 1.21 nmol/ml for those with TC genotype, and 1.22 nmol/ml for those with CC genotype (p for trend = 0.33).

**Table 2:** Geometric means for TBARS by *MnSOD*, *GSTP1*, *GSTM1*, *GPX1*, *GPX3*, and *CAT* genotypes, WEB study controls, Erie and Niagara counties, 1996–2001^a^.

Genotype	All participants, n = 1402		Premenopausal, n = 447		Postmenopausal, n = 955	
	n	Geometric means (95% CI)^b^	n	Geometric means (95% CI)^b^	n	Geometric means (95% CI)^b^
***MnSOD***						
TT	348	1.35(1.31–1.39)	117	1.25(1.19–1.31)	231	1.40(1.35–1.45)
TC	682	1.28(1.25–1.31)	222	1.21(1.17–1.25)	460	1.32(1.29–1.35)
CC	372	1.31(1.27–1.34)	108	1.22(1.16–1.28)	264	1.34(1.30–1.39)
p for trend		0.01		0.33		0.015
***GSTP1***						
AA	661	1.31(1.28–1.33)	222	1.21(1.17–1.25)	439	1.35(1.32–1.39)
AG	593	1.31(1.28–1.33)	182	1.25(1.20–1.29)	411	1.34(1.30–1.37)
GG	148	1.29(1.24–1.35)	43	1.19(1.10–1.29)	105	1.34(1.27–1.41)
p for trend		0.91		0.34		0.71
***GSTM1***						
Not null	584	1.31(1.28–1.34)	192	1.25(1.21–1.30)	392	1.34(1.30–1.37)
Null	818	1.30(1.28–1.32)	255	1.20(1.16–1.24)	563	1.35(1.32–1.38)
p for trend		0.52		0.07		0.60
***GPX1***						
CC	654	1.31(1.28–1.33)	204	1.20(1.16–1.25)	450	1.36(1.32–1.39)
CT	621	1.30(1.27–1.33)	199	1.24(1.19–1.28)	422	1.33(1.30–1.36)
CC	127	1.32(1.26–1.38)	44	1.24(1.15–1.34)	83	1.36(1.29–1.44)
p for trend		0.79		0.53		0.49
***GPX3***						
AA	1019	1.31(1.28–1.32)	331	1.22(1.19–1.26)	688	1.34(1.32–1.37)
AC	355	1.31(1.27–1.34)	109	1.22(1.16–1.28)	246	1.35(1.30–1.40)
CC	28	1.29(1.17–1.42)	7	1.10(0.90–1.33)	21	1.38(1.23–1.54)
p for trend		0.95		0.56		0.88
***CAT***						
CC	848	1.31(1.28–1.33)	273	1.24(1.20–1.28)	575	1.34(1.31–1.37)
CT	491	1.31(1.27–1.34)	151	1.21(1.16–1.26)	340	1.35(1.32–1.39)
TT	63	1.24(1.17–1.33)	23	1.09(0.98–1.22)	40	1.33(1.23–1.45)
p for trend		0.36		0.07		0.80

^a^TBARS- Thiobarbituric acid-reactive substances

^b^ adjusted for age

SNPs in the other genes examined (*GSTP1*, *GSTM1*, *GPX1*, *GPX3*, and *CAT*) were not associated with TBARS, either in the overall group or in strata defined by menopausal status.

## Discussion

In a cross-sectional analysis, we found that genetic variation in *MnSOD* is associated with a blood marker of oxidative stress, particularly among postmenopausal women. There were no associations for the other SNPs studies for genes which play a role in control of oxidation.

Manganese superoxide dismutase is located in the mitochondria, converting the superoxide anion radical resulting from the electron transport chain into oxygen and hydrogen peroxide [3]. It has been hypothesized that the Ala (C-allele) variant may favor easier transport of the MnSOD precursor into the mitochondria [8] and may result in higher basal activity of the enzyme [37]. The Val-containing variant (T-allele), on the contrary, might become partially arrested within the inner mitochondria membrane [8] impacting its function. However, in one study it was found that individuals homozygous for the C-allele have lower MnSOD enzyme activity compared to those with TC or TT genotypes [12]. Some studies have shown an association of the C-allele with increased risk of certain cancers, particularly in homozygous individuals, compared to the individuals homozygous for the T-allele [13–17]. Our results suggest that heterozygous individuals may have the greatest ability to cope with oxidative stress; those with the TT genotype the lowest.

There is little previous research regarding the association between genetic variants and oxidative stress biomarkers in a healthy population, and findings have not been consistent. Hong et al. found that having at least one C-allele was associated with higher urine levels of 8-OH-dG, a measure of oxidative DNA damage, compared to those homozygous for the T-allele [24]. It should be noted, however, that the study’s sample was small (n = 81) and consisted of term pregnant women. Our sample consisted of non-pregnant women, and was larger. We found that the association with *MnSOD* was limited to postmenopausal women. Menopause is characterized by the drop of production of estrogens, hormones that can act as antioxidants [38, 39]. This loss of antioxidant defense by estrogen may result in higher oxidative stress among postmenopausal women when compared to premenopausal women [38]. Premenopausal women remain protected from oxidative stress by these hormones. In our study, the lack of an association of the *MnSOD* genotype with TBARS among premenopausal women may be related to their higher concentrations of circulating estrogen and estrogen metabolites, such that the impact of genotype was not detectable.

Understanding of the strengths and limitations of this study are important in analysis of the findings. There are some concerns with TBARS as a biomarker of oxidative status. TBARS is not completely specific to malondialdehyde (MDA) and can be affected by reaction of the assay with other compounds [3, 33]. These compounds may originate during lipid peroxidation processes and may also impact the results of the test [40]. MDA in the sample can also be formed through other mechanisms, not exclusively by lipid peroxidation [32]. Despite these concerns, however, TBARS is a useful indicator of overall oxidative stress [33]. In fact, it has been found to be elevated among cancer patients, individuals with an excessive oxidative stress burden, compared to controls [41, 42].

Moreover, we examined a group of SNPs because of information regarding their functional impact on the corresponding enzyme. It could be that other genetic variants in those genes or other related genes are causally associated with TBARS. Given the complexity of the processes to maintain oxidative balance, these analyses address only part of that process. Finally, oxidative stress measured in blood may not be representative of reactions that take place on cellular and sub-cellular levels. There might be different, even stronger associations with measures of oxidative status in tissues. Further, the finding of an association of TBARS with *MnSOD* could be the result of multiple testing. Further exploration of these associations in other samples would be important in determining the consistency of the association.

This study does have some important strengths. These include the large sample size (n = 1402) which allowed us to examine subgroups defined by menopausal status. The population-based sample is also an advantage, allowing for external validity of the findings.

In conclusion, we found that oxidative stress may be associated with genetic variation in *MnSOD*, particularly among postmenopausal women. Understanding of the factors both endogenous and exogenous which impact oxidative status is critical to understanding oxidation in chronic disease processes such as cardiovascular diseases and cancers. Our observations need further exploration in order to better understand the role of ROS in the pathogenesis of those chronic diseases.
